# A comparative study of anhedonia components between major depression and schizophrenia in Chinese populations

**DOI:** 10.1186/s12991-015-0061-3

**Published:** 2015-09-03

**Authors:** Yinghui Li, Xiaodong Mou, Wenhao Jiang, Zhong Yang, Xinhua Shen, Zhuma Jin, Zhiping Dai, Yuju Liu, Shengqin Mao, Jian Zhang, Yonggui Yuan

**Affiliations:** Department of Psychosomatics and Psychiatry, ZhongDa Hospital, School of Medicine, Southeast University, Nanjing, 210009 People’s Republic of China; Department of Psychiatry, Changshu Mental Health Centre, Changshu, China; Department of Psychosomatics, The Third People’s Hospital of Huzhou, Huzhou, China; Department of Psychiatry, Brain Hospital affiliated to Nanjing Medical University, Nanjing, China; Department of Psychiatry, The Fourth People’s Hospital of Zhenjiang, Zhenjiang, China; Department of Psychiatry, The Third People’s Hospital of Haian, Haian, China

**Keywords:** Anhedonia, Anticipatory pleasure, Consummatory pleasure, Major depressive disorder, Schizophrenia

## Abstract

**Background:**

Anhedonia is a prominent symptom of major depressive disorder (MDD) and schizophrenia. At present, it is believed that hedonic processing rather consists of the anticipatory and consummatory phase. The aim of this research is to explore the different anhedonia components in MDD and schizophrenia in Chinese populations.

**Methods:**

A Chinese version of the Temporal Experience of Pleasure Scale (TEPS) was used to evaluate 176 MDD patients, 346 schizophrenia patients, and 268 healthy controls. Additionally, the 17-item Hamilton Depression Rating Scale (HAMD-17) was used for MDD patients, while the Positive and Negative Syndrome Scale (PANSS) was applied for schizophrenia.

**Results:**

The scores of consummatory (TEPS-CON) and anticipatory pleasure (TEPS-ANT) in MDD and schizophrenia were both significantly lower than healthy controls (both P < 0.001). TEPS-CON and TEPS-ANT were negatively correlated with the score of HAMD-17, the duration of illness and admission times in MDD (P < 0.05 or 0.01). TEPS-CON was negatively related to PANSS total scores and negative symptoms (P < 0.05 or 0.01), but no significant correlation was found with duration of illness and admission times in schizophrenia (P > 0.05). There was no significant correlation between TEPS-ANT and any clinical variables (P > 0.05).

**Conclusions:**

The consummatory and anticipatory pleasures were both impaired in MDD and schizophrenia. Consummatory and anticipatory anhedonia can be considered as a “state” in MDD, but as a “trait” in schizophrenia.

## Background

Anhedonia, the diminished capacity to experience pleasure, is a prominent symptom of major depressive disorder (MDD), schizophrenia and other neuropsychiatric disorders [1–4]. Almost 30 years ago, Klein [5] suggested that hedonic processing was not a unitary construct, but rather a complex of both anticipatory and consummatory components. Consummatory pleasure reflects the momentary pleasure that is experienced while engaged in an enjoyable activity, while anticipatory pleasure revolves around pleasure from future activities.

Anhedonia and depressed mood are the two core symptoms of MDD [1]. Hasler and colleagues [6] demonstrated that anhedonia together with increased stress reactivity was the most important candidate for psychopathological endophenotype of MDD. Joiner et al. [7] found that MDD patients presented higher scores on Beck Depression Inventory (BDI) anhedonic items than schizophrenic subjects, suggesting that anhedonia was a specific state-like feature of depressive illness. However, Pelizza and Ferrari [8] detected that anhedonia reached highly significant levels in 36.9 % of depressed patients and 45 % of schizophrenic patients according to the Physical Anhedonia Scale and the Social Anhedonia Scale, indicating that anhedonia could not be considered as a distinctive state in MDD.

Accumulating evidence suggested that depression was associated with anhedonia. Pizzagalli et al. [9] found blunted reward responsiveness in patients with MDD, particularly when anhedonic symptoms were prominent. Treadway et al. [10] observed an association between anhedonia and reduced motivation in an effort-based decision-making task in MDD. Liu et al. [11] utilizing the Snaith–Hamilton Pleasure Scale (SHAPS) in Chinese populations indicated that patients with depression demonstrated more anhedonic symptoms than patients with schizophrenia and non-clinical individuals. Although the above studies suggested diminished hedonic capacity in MDD, they did not differentiate consummatory and anticipatory anhedonia. To date, only one study distinguished consummatory and anticipatory anhedonia, stating low anticipatory pleasure but intact consummatory pleasure in depressed individuals [12].

In schizophrenia, anhedonia has long been considered a cardinal symptom [13] and a component of negative symptoms [14, 15]. Studies on anhedonia in schizophrenia have provided inconsistent results. Schizophrenia patients reported more severe anhedonia than healthy controls on self-report measures and semistructured interviews [16–18], but had normal levels of pleasant emotions while in response to pleasurable stimuli [19, 20]. Distinction of deficits in anticipatory and consummatory pleasure may help to understand the mixed findings on anhedonia in schizophrenia. A new self-report questionnaire, the Temporal Experience of Pleasure Scale (TEPS), developed by Gard et al. [21], was used to assess consummatory and anticipatory pleasure. Several studies showed anticipatory, but not consummatory anhedonia in schizophrenia [22–24]. However, the study of Strauss et al. [25] had the opposite result. Buck and Lysaker [26] suggested that anticipatory pleasure predicted concurrent and prospective levels of positive symptoms, emotional discomfort and interpersonal function, whereas consummatory pleasure predicted only concurrent positive symptoms.

In summary, little attention has been paid to detect the consummatory and anticipatory pleasure impairment in MDD, and there were inconsistent results on consummatory and anticipatory pleasure in schizophrenia. Moreover, there has been no study to compare the difference between MDD and schizophrenia on consummatory and anticipatory pleasure.

In this study, we assessed consummatory and anticipatory pleasure using TEPS in MDD and schizophrenia. The present study has two main aims: (1) examining the discrimination of consummatory and anticipatory pleasure between MDD or schizophrenia and healthy controls; and (2) exploring the relationships between different anhedonia types and clinical variables, including severity, duration of illness and admission times in MDD and schizophrenia.

## Methods

### Participants

This study included 176 patients with MDD, 346 patients with schizophrenia and 268 healthy controls. All the patients were inpatients who were recruited from six hospitals (one general hospital, five psychiatric hospitals): Zhongda Hospital affiliated to Southeast University, Changshu Mental Health Centre, Third People’s Hospital of Hai’an, Brain Hospital affiliated to Nanjing Medical University, Fourth People’s Hospital of Zhenjiang and Third People’s Hospital of Huzhou. Clinical diagnoses were determined based on the Structured Clinical Interview for DSM-IV [1] by experienced psychiatrists. Patients with any other concurrent Axis I disorders were excluded; furthermore, patients with history of mental retardation, personality disorder, neurological disorders, such as stroke, head injury, seizure or diagnosis of alcohol or substance abuse/dependence in the last 6 months were also excluded from the study. All patients with MDD received antidepressant medication treatment, tricyclic antidepressants (TCAs, n = 12), selective serotonin reuptake inhibitors (SSRIs, n = 86), serotonin–norepinephrine reuptake inhibitors (SNRIs, n = 43), noradrenergic and specific serotonergic antidepressant (NaSSA, n = 25), other antidepressants (n = 10) and two or more antidepressants (n = 12). All patients with schizophrenia were treated with a regular dose of antipsychotic medications: typical (n = 42), atypical (n = 274) and mixed (n = 30). Healthy controls were recruited from ZhongDa Hospital, including 216 health examination clients, 32 nurses and 20 doctors. All healthy controls were screened by experienced psychiatrists to ascertain that they did not have any neuropsychiatric symptoms.

### Measures

#### General Situation Questionnaire

A self-designed questionnaire was used to investigate the general condition of participants, including demographical information, course of illness, admission times and type of medications.

#### The Temporal Experience of Pleasure Scale (TEPS)

The TEPS is composed of 18 items rated on a Likert-type scale ranging from 1 (Very True for me) to 6 (Very False for me) and yields two subscales measuring anticipatory (TEPS-ANT) and consummatory (TEPS-CON) pleasure. Lower scores indicate greater levels of anhedonia [21]. The Chinese version of TEPS has been proved to possess adequate validations and reliability in previous studies [24, 27]. The current study found that TEPS-ANT and TEPS-CON were positively correlated with the General Self-efficacy Scale [28] (r = 0.27, 0.25, separately, P < 0.01) and inversely related with the BDI [29] (TEPS-ANT r = −0.19, −0.13, separately, P < 0.01). The correlation between the TEPS and other scales suggested that the TEPS had adequate convergent and discriminant validity. In this study, the Chinese version of TEPS had demonstrated good internal consistency (TEPS-ANT, alpha = 0.706; TEPS-CON, alpha = 0.702) and test–retest reliability (TEPS-ANT, r = 0.698; TEPS-ANT, r = 0.615).

#### 17-item Hamilton Depression Rating Scale (HAMD-17)

Depression severity was measured with the Chinese version HAMD-17 in depression patients. A baseline HAMD-17 score ≥17 was applied as an inclusion criterion. The Chinese version of HAMD-17 has demonstrated good internal consistency and test–retest reliability [30].

#### Positive and Negative Syndrome Scale (PANSS)

PANSS was applied to evaluate the clinical severity of positive, negative and other symptoms in schizophrenia. It is a 30-item scale, in which 7 constitute a positive scale, 7 a negative scale and the remaining 16 a general psychopathology scale. Each item contains seven rating points representing increasing levels of psychopathology. The Chinese version of PANSS has demonstrated good internal consistency (alpha 0.73-0.83 depending on the sub-scale) [31].

### Procedure

All participants for this study filled out the TEPS. Additionally, patients with major depression completed HAMD-17 to measure depression severity, and patients with schizophrenia completed PANSS to measure clinical symptoms. All participations were given written informed consent to participate in the study. The study procedure was approved by the Ethical Committee of ZhongDa Hospital affiliated to Southeast University, China.

### Statistical analyses

Categorical variables were compared by means of the Chi-square test. One-way analysis of variance (ANOVA) was employed to compare continuous variables among groups, and post hoc LSD tests were performed in cases of significant ANOVA effects. Pearson correlations were conducted to assess relationships between anhedonia and clinical variables. Statistical analyses were performed using the SPSS program version 11.5.

## Results

### Participants’ demographic and clinical characteristics

The participants’ demographic and clinical characteristics are reported in Table 1. There was no significant difference among these three groups in gender(χ2 = 2.01, *P* > 0.01), age (F = 1.73, *P* > 0.01) and the level of education (F = 1.75, *P* > 0.01).

**Table 1:** Socio-demographic and clinical characteristics of the study population

	Patients with MDD (*n* = 176)	Patients with schizophrenia (*n* = 346)	Heathy controls (*n* = 268)
Age (year)	35.51 ± 11.88	33.75 ± 10.22	34.10 ± 9.47
Females/males	98/79	178/168	135/133
Education (year)	10.86 ± 3.01	10.62 ± 2.48	11.08 ± 3.64
Duration of illness (year)	5.71 ± 8.98	8.10 ± 8.79	–
Admission times	2.20 ± 1.82	3.53 ± 3.31	–
HAMD-17	16.12 ± 8.05	–	–
PANSS total	–	69.09 ± 22.04	–
PANSS positive	–	16.70 ± 7.40	–
PANSS negative	–	18.04 ± 6.98	–
PANSS general	–	33.69 ± 11.11	–

### Comparisons between patients with MDD or schizophrenia and healthy controls

**Fig. 1:**
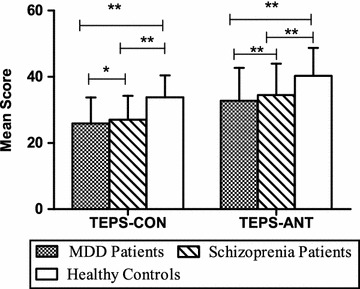
Comparisons of TEPS-CON and TEPS-ANT between patients with MDD or schizophrenia and healthy controls. *P < 0.05,**P < 0.01. One-way ANOVA indicated significant differences in the TEPS-ANT and TEPS-CON scores (both *P* < 0.001) among the three groups. Post hoc tests showed that TEPS-ANT and TEPS-CON scores in MDD and schizophrenia were both lower than those in healthy controls (all *P* < 0.01), and TEPS-ANT and TEPS-CON scores in MDD were lower (*P* < 0.01 or 0.05 respectively) than those in schizophrenia

One-way ANOVA indicated significant differences in the TEPS-ANT and TEPS-CON scores (F = 40.8, 89.1 respectively, *P* < 0.001) among the three groups. Post hoc tests showed that TEPS-ANT and TEPS-CON scores in MDD and schizophrenia were both lower than those of healthy controls (all *P* < 0.01), and TEPS-ANT and TEPS-CON scores in patients with MDD were lower (*P* < 0.01 or 0.05, respectively) than in those with schizophrenia (Fig. 1).

### Associations between TEPS subscales and clinical variables in patients with MDD

Table 2 presents that both TEPS-CON and TEPS-ANT scores were negatively correlated with HAMD-17 scores, duration of illness and admission times in patients with MDD (*P* < 0.05 or 0.01).

**Table 2:** The Pearson correlation coefficients between TEPS and clinical characteristics in patients with MDD

	TEPS-CON	TEPS-ANT
HAMD-17	−0.48**	−0.54**
Duration of illness (years)	−0.18*	−0.21**
Admission times	−0.21**	−0.17*

* *P* < 0.05

** *P* < 0.01

### Associations between TEPS subscales and clinical variables in patients with schizophrenia

As shown in Table 3, TEPS-CON scores were inversely correlated with PANSS total scores, and positive and negative symptoms (*P* < 0.05 or 0.01) in schizophrenia. There was no significant correlation between TEPS-CON and general psychopathology, duration of illness or admission times (*P* > 0.05). TEPS-ANT showed no significant correlation with all clinical characteristics (PANSS total scores, positive symptoms, negative symptoms, general psychopathology, duration of illness and admission times) in schizophrenia patients.

**Table 3:** The Pearson correlation coefficients between TEPS subscales and clinical characteristics in patients with schizophrenia

	TEPS-CON	TEPS-ANT
PANSS total	−0.14**	−0.07
PANSS positive	−0.11*	−0.09
PANSS negative	−0.78**	0.06
PANSS general	−0.10	0.07
Duration of illness (years)	−0.004	−0.08
Admission times	0.03	−0.08

* *P* < 0.05

** *P* < 0.01

## Discussion

The present study indicated that both consummatory and anticipatory pleasure were impaired in MDD. The results were not completely consistent with most previous studies which usually reflected either consummatory anhedonia [7, 8, 11] or anticipatory pleasure impaired by depression [12]. It is noteworthy that several theorists have proposed that anhedonic features are related to altered dopaminergic circuitry in MDD [32–34]. Particularly, the dysfunction of dopaminergic mesolimbic and mesocortical reward circuits has been singled out as the major neurobiological correlate of anhedonia [35, 36]. Mesolimbic dopaminergic neurons projecting into the frontal cortex and nucleus accumbens have been suggested to be involved in the therapeutic actions of some antidepressants [37, 38], through 5-HT modulation of DA function [39]. Previous studies have shown that antidepressants can influence anhedonia in patients with MDD in dopaminergic mesolimbic reward circuits likely caused by the increase in serotonin neurotransmission [38, 40, 41]. We speculate that the difference between the present study and other studies may be due to the influence of antidepressants on anhedonia.

This study also found anticipatory and consummatory pleasure impaired in schizophrenia. Gard et al. [22] and Chan et al. [24] observed lower anticipatory pleasure in schizophrenia compared with healthy controls. But Strauss et al. [25] found lower score on consummatory pleasure. Our findings were not completely consistent with the mentioned studies. Strauss et al. [25] supposed that the main difference between their study and the study of Gard et al. and Chan et al. was in the percentage of patients who were prescribed typical antipsychotics. We agreed with this speculation. In the present study, 21 % of patients were prescribed typical antipsychotics, compared to 31 % in Gard et al. 29 % in Chan et al. and 12 % in Strauss et al. Previous evidence showed that typical antipsychotics could produce anhedonia via dopamine antagonism [42], and a neurobiological study showed that dopamine was strongly linked to anticipatory rather than consummatory pleasure [43, 44]. In contrast, consummatory pleasure was more strongly linked to the serotonin and opioid systems [45, 46]. Further evidence also revealed that typical antipsychotics lead to more reward prediction dysfunction [47], and therefore anticipatory rather than consummatory pleasure was even more easily influenced by typical antipsychotics. Another reason for this might be that previous studies missed some factors such as relapse times, acute or chronic phase and patients in hospitals or community homes [22, 24, 25].

In the correlation study, we found diminished consummatory and anticipatory pleasure associated with depression severity in patients with MDD. The results were in concordance with other studies showing that the severity of anhedonia was positively correlated with the severity of depressive symptoms in MDD [48–52]. We also found that TEPS-CON and TEPS-ANT had significant positive correlations with the duration of illness and admission times in MDD. The results indicated that impaired consummatory and anticipatory pleasure resulted in worse prognosis of MDD. It was consistent with the opinion that anhedonia was a post-depressive “scar” symptom in MDD [53].

In previous studies, anhedonia did not show strong association with the duration of illness, number of admissions, cumulative time in hospital and length of current admission in schizophrenia. It remains fairly stable over time and is generally considered to reflect as a “trait” rather than a “state” in schizophrenia [15, 54, 55]. However, whether anticipatory and consummatory anhedonia would be stable over time in schizophrenia was unclear. The current results indicated that TEPS-ANT and TEPS-CON scores had no significant relationship with the course of illness and admission times, which suggested that both anticipatory and consummatory anhedonia remained relatively stable in schizophrenia.

Herbener and Harrow found significant correlations between anhedonia and negative symptoms, but no meaningful relationships with positive symptoms in schizophrenia in a 10-year longitudinal study [56]. However, other studies indicated significant associations between anhedonia and positive symptoms [57,58]. No statistically significant associations between anhedonia and positive, negative and general psychopathology scores as measured by PANSS were detected in the research of Huxley et al. [59]. In this study, we found that TEPS-CON significantly correlated with PANSS total scores, negative and positive symptoms, but TEPS-ANT had no meaningful relationship with any symptoms. The results suggested that consummatory anhedonia might vary with the severity of negative and positive symptoms, but anticipatory anhedonia seemed to be independent of many clinical variables, such as positive and negative symptoms. This seemingly showed that anticipatory anhedonia was more stable than consummatory pleasure deficit in patients with schizophrenia.

There were some limitations in this study. First, the sample of patients was recruited from clinical randomly as long as the patients met the inclusion criteria. All the patients in our study were taking medication; although the differences among medications were taken into account, we did not consider the influence of different doses on anhedonia in patients with MDD and schizophrenia. Second, different sites and inter-rater bias could influence the measurement results. Finally, this study did not measure TEPS in different clinical phases to understand whether consummatory and anticipatory pleasure would vary with time.

## Conclusions

Consummatory and anticipatory anhedonia were both found in MDD and schizophrenia. The two anhedonia components were significantly associated with depression severity, duration of illness and admission times in MDD. Therefore, we speculated that both consummatory and anticipatory pleasure deficit could be regarded as a “state” in MDD. However in schizophrenia, consummatory anhedonia was only associated with negative and positive symptoms, and anticipatory anhedonia had no significant correlation with any clinical characteristics. So, anticipatory and consummatory anhedonia can be considered as a “trait”, and it seemed more stable than consummatory anhedonia in schizophrenia.

## Abbreviations

DSM-IV: Diagnostic and Statistical Manual of Mental Disorders Criteria-fourth edition
SHAPS: Snaith–Hamilton Pleasure Scale
BDI: Beck Depression Inventory
TEPS: Temporal Experience of Pleasure Scale
TEPS-CON: consummatory pleasure in Temporal Experience of Pleasure Scale
TEPS-ANT: anticipatory pleasure in Temporal Experience of Pleasure Scale
HAMD-17: 17-item Hamilton Depression Rating Scale
PANSS: Positive and Negative Syndrome Scale
